# Numerical Activities and Information Learned at Home Link to the Exact Numeracy Skills in 5–6 Years-Old Children

**DOI:** 10.3389/fpsyg.2016.00094

**Published:** 2016-02-11

**Authors:** Silvia Benavides-Varela, Brian Butterworth, Francesca Burgio, Giorgio Arcara, Daniela Lucangeli, Carlo Semenza

**Affiliations:** ^1^Department of Developmental Psychology and Socialization, University of PadovaPadova, Italy; ^2^Institute of Cognitive Neuroscience and Psychology Department, University College LondonLondon, UK; ^3^Neuropsychology Unit, Istituto di Ricovero e Cura a Carattere Scientifico San Camillo Hospital FoundationLido-Venice, Italy; ^4^Neuroscience Department, University of PadovaPadova, Italy

**Keywords:** early numeracy, activities within the family environment, numerical information learned at home, exact representations, approximate representations, board games

## Abstract

It is currently accepted that certain activities within the family environment contribute to develop early numerical skills before schooling. However, it is unknown whether this early experience influences both the exact and the approximate representation of numbers, and if so, which is more important for numerical tasks. In the present study the mathematical performance of 110 children (mean age 5 years 11 months) was evaluated using a battery that included tests of approximate and exact numerical abilities, as well as everyday numerical problems. Moreover, children were assessed on their knowledge of number information learned at home. The parents of the participants provided information regarding daily activities of the children and socio-demographic characteristics of the family. The results showed that the amount of numerical information learned at home was a significant predictor of participants' performance on everyday numerical problems and exact number representations, even after taking account of age, memory span and socio-economic and educational status of the family. We also found that particular activities, such as board games, correlate with the children's counting skills, which are foundational for arithmetic. Crucially, tests relying on approximate representations were not predicted by the numerical knowledge acquired at home. The present research supports claims about the importance and nature of home experiences in the child's acquisition of mathematics.

## Introduction

At the time children enter school education they already show great individual differences in their numerical performance (e.g., Aunola et al., 2004). There are three reasons why this may be so. First, general cognitive factors, such as intelligence, working memory capacity, and so on, could differentiate individual learners (e.g., Espy et al., 2004; Gathercole et al., 2004; Bull et al., 2008; Passolunghi et al., 2008; Kroesbergen et al., 2009; Geary, 2011; Passolunghi and Lanfranchi, 2012). Second, cognitive factors specific to the domain of numbers could be critical (e.g., Aunola et al., 2004; Siegler and Booth, 2004; Booth and Siegler, 2006; Halberda et al., 2008; Butterworth, 2010; Reeve et al., 2012). Third, contextual factors, such as social, economic, parental influences, could play the key role. Of course, all these factors interact and it is difficult to determine their influence separately or indeed together (Butterworth, 2005). Here we focus on one contextual factor, numerical activities and knowledge acquired in the learner's home. In particular, we seek to assess the effects of numerical activities and the information learned within the family environment on two potential types of domain-specific capacities that we inherit: the Approximate Number System (e.g., Carey, 2004, 2009; Feigenson et al., 2004) and the Exact Number System (e.g., Gelman and Gallistel, 1986; Butterworth, 2010).

Parents usually report using literacy activities (e.g., sharing book reading) more frequently than numeracy activities with their children at home (Blevins-Knabe et al., 2000; Cannon and Ginsburg, 2008; LeFevre et al., 2009). The implementation of reading-related practices in family settings has been reinforced in the last decades by strong recommendations conveyed through the media and schools, which in turn rely on the findings of numerous studies on home literacy and the acquisition of reading skills (see Fletcher and Reese, 2005, for a review). Similar contributions for promoting numerical practices at home are just being explored (Berkowitz et al., 2015), which call for further studies aiming at understanding how mathematical knowledge can be acquired outside of school.

### Contextual factors influencing arithmetical attainment

Parents' expectations about numeracy play a significant role in the basic calculation skills of their children. One longitudinal study showed that children's attitudes toward mathematics were influenced more by their parents' beliefs about their child's abilities than by the child's own results in previous mathematical assessments (Parsons et al., 1982). Sheldon and Epstein (2005) showed that parents who implemented specific practices to support math learning at home (e.g., discussing the student's homework, lending library activities, or playing games by indication of the experimenters) contributed to increasing the children's scores on math achievement tests. Kleemans et al. (2012) found similar results: the higher the parents' numeracy expectations, the better the child's early numeracy skills.

The socioeconomic background of the family also influences arithmetical development (Melhuish et al., 2008). A series of studies by Jordan a collaborators show that children from impoverished backgrounds display poorer numerical capacities than peers from advantaged backgrounds in tasks such as counting, adding, subtracting, and comparing magnitudes (Jordan et al., 1994, 2006). Similar conclusions were reached in a cross-cultural study derived from the Program for International Student Assessment–PISA—(Ming Chiu and Xihua, 2008). In the study, the authors evaluated the mathematical performance of more than one hundred thousand children from 41 countries and explored whether their scores related to characteristics of the family of origin. Consistent with previous evidence, it was reported that children who scored highest in the tests came from families that had more cultural possessions and higher socio-economical status.

### The influence of home numeracy learning experiences

One potential causal factor in the above studies is that socially more advantaged parents with higher numeracy expectations engaged in more numeracy-related practices, which in turn is associated with children's higher mathematics achievements (LeFevre et al., 2010). The pioneer study of Blevins-Knabe and Musun-Miller (1996) investigated the frequency of specific child and parent-child activities at home that directly involved the use of numbers. The results showed a positive correlation between activities that reflect direct number instruction (i.e., does the child say the words “one,” “two,” “three,” or does the child mention number facts such as 1 + 1 = 2) and children's total scores on the standardized test of Early Mathematics Ability-Second Edition (TEMA-2 of Ginsburg and Baroody, 1990). Similarly, Huntsinger et al. (2000) found a positive correlation between the parents' deliberate efforts to teach their children simple sums and the children's later achievement in basic calculation skills. Moreover, LeFevre and colleagues found that the frequency of parent–child number teaching activities (i.e., counting, simple addition) was directly related to the counting abilities of their children (LeFevre et al., 2002). Analogous results were found for Chinese-speaking children: the frequency of parent–child numeracy activities was significantly related to later performance on counting and addition (Pan et al., 2006).

The above studies consistently show a relation between direct numerical instruction at home and children's math performance. With respect to the relations between numeracy knowledge and other daily activities that indirectly facilitate acquisition, the literature is much less developed. LeFevre et al. (2009) first proposed the distinction between direct and indirect numerical activities within the family environment. Direct activities are those typically used by parents for the explicit purpose of developing quantitative skills (e.g., counting objects, practicing number names, printing numbers). In contrast, indirect activities are real-world tasks (e.g., playing card or board games) for which the acquisition of numeracy is likely to be incidental. The crucial distinction is that, although instruction in numeracy occurs in both types of activities, teachings are embedded in everyday tasks only in indirect numerical activities. In adults, recent studies have shown that formal mathematical performance and the use of numerical information in everyday activities might dissociate both at the behavioral (Semenza et al., 2014) and the neural level (Benavides-Varela et al., 2015). In children, to the best of our knowledge, only one study has been conducted so far showing how indirect experiences at home relate to children's quantitative skills. In particular, LeFevre et al. (2009) evaluated the relation between the frequency of home numeracy activities and children's performance on three subtests of the KeyMath test: addition, subtraction and numeration. The authors found a positive correlation between children's performance on the KeyMath and their frequency of participation in board games. Interestingly, the accuracy and latency of the children's responses also correlated with the frequency with which the parents reported using the calendars and dates, and talking about money within the family environment. Unfortunately this study, like previous ones, based its conclusions on single scores of performance tests (e.g., KABC battery in Anders et al., 2012; TEMA-2 in Blevins-Knabe and Musun-Miller, 1996) thus leaving unresolved the question of whether the activities within the family environment differentially affect specific mathematical skills and critically whether those skills rely on either the exact number system, or the approximate numerical system, or both.

In agreement with the study of LeFevre and colleagues, Ramani and Siegler (2008) showed that playing linear number board games at home influences numerical knowledge in early childhood. However, instead of interviewing parents, the authors asked the children about the frequency and number of board games, card games, and videogames they played outside the school. The authors found a positive correlation between the amount of board game experience and the performance on four numerical tasks: numerical magnitude comparison, number line estimation, counting, and numeral identification. This study suggests that playing linear board games enhances the performance on tasks relying on both exact and approximate representations. However, this result was found only in a portion of the population under study whereas for the remaining kids, namely those from high socio-economical status, the association between performance on the tests and the frequency of playing board games was not found. Moreover, this study focused only on a single activity. It is thus unclear whether other activities within the family environment influence numerical understanding and to what extent they differentially impact exact and approximate systems.

### The present study

Whilst the ability to roughly approximate the numerosities of sets is present in humans even from birth (Izard et al., 2009), the exact representation of numbers appears to develop later in life either by “bootstrapping” approximate into exact representations (Carey, 2004, 2009) or independently by mapping onto number words in the process of learning to count (Gelman and Gallistel, 1986; Leslie et al., 2008). It has been observed that formal education enhances the acuity of the exact, as well as the approximate representations (Piazza et al., 2013); and crucially that young children's skills on both approximate (e.g., Booth and Siegler, 2008; De Smedt et al., 2009; Mazzocco et al., 2011; Desoete et al., 2012) and exact representations (e.g., Geary et al., 1992; Aunola et al., 2004; Passolunghi et al., 2007; Reeve et al., 2012) are excellent long term predictors of later school mathematical performance. However, it is currently unknown to what extent the exact and approximate skills are also influenced by the activities and the numerical knowledge acquired in the household environment. Because home experiences often directly or indirectly imply sequencing and naming numbers (LeFevre et al., 2009), they are likely to influence tasks that rely on exact number knowledge. However, it is also possible that numerical activities within the family environment and knowledge acquire at home impact tasks relying on approximate representations, either by directly triggering changes in those representations (Ramani and Siegler, 2008) or through a connection between the exact and approximate systems. The last possibility gets support by work showing that children who know more number words and Arabic numbers also perform better in tasks relying on approximate representations (Mussolin et al., 2012).

In the present work, we thus aimed at studying the effects of indirect numerical exposure further, focusing on how activities within the family environment and the numerical information learned at home relate to each of the representational systems underlying mathematical performance. This information should contribute to understand the permeability of the exact and the approximate systems in early childhood and should consequently be useful to elaborate refined educational programs adapted to the needs of the learners who begin the transition from the family to the pre-school/school learning environments. The tasks implemented in the current study are well known in the field. They were adopted from established groups that have evaluated approximate representations in magnitude comparison (Halberda et al., 2008) and the number line tasks (Siegler and Booth, 2004); and exact representations such as counting (Geary et al., 1992) and one-to-one correspondence (Van Luit et al., 1994).

The current work is different from the previous studies in several respects. First and foremost: the abilities under investigation. The most influential study that has so far focused on the effects of indirect exposure to numbers in the family environment measured arithmetical performance (LeFevre et al., 2009). Our study additionally incorporates tests of approximate representations and crucially also of numerical problems in everyday situations. We reasoned that evaluating abstract arithmetic abilities (e.g., 1 + 3 = ?) in young children might provide an incomplete measure of their early mathematical intuitions because children are generally unfamiliar with the formal terms “plus,” “minus,” or “equals” before entering school. Measuring the same abilities applied to everyday situations (e.g., if you have one banana and I give you three more, then how many bananas do you have now?) should provide a clearer view of the children arithmetic intuitions and the way they interact with the numerical exposure received at home. Moreover, the present study incorporates a direct measure of the child knowledge of number facts that are learned through parental or familiar instruction (e.g., birthdates, phone numbers, etc.). Previous studies based their conclusions on parents' reports of family activities and not on the actual knowledge of the child. The current measure, which we call “numerical information learned at home” directly inquires children numerical facts that are acquired within the family. It should therefore provide a closer approximation to the actual opportunities children have to learn this information. Finally, our study differs from previous ones in the sources of information regarding the specific activities and the frequency of those activities within the family environment. Specifically, while previous studies focused either on parental report (Parsons et al., 1982; Sheldon and Epstein, 2005; LeFevre et al., 2009), or an interview for children (Ramani and Siegler, 2008), the present study integrates these two approaches. Children accounts have the advantage of capturing information that parents may overrate or miss (Tudge and Doucet, 2004). Parental accounts, on the other hand, are more stable and less influenced by preferences or recent memories. Therefore, combining the information provided by parents and children and evaluating the consistency between them should offer a more reliable account than the one provided by each of these informants separately.

## Methods

### Participants

A hundred and ten pre-school children (59 females; 100 right-handers; mean age: 5.95 years-old; age range: 5.46–6.43 years-old) participated in the study. Participants were enrolled in the last year of kindergarten in five different urban and extra-urban schools of the province of Padua, Italy. The schools and teachers involved in the research were contacted in a meeting of Educational Psychology organized by the University. All children had normal or corrected-to-normal eyesight and their parents reported no history of health problems during pregnancy, infancy, or childhood that would compromise their perceptual or intellectual abilities. Direct measures of intellectual abilities were not obtained because the Italian policies allow them only for clinical purposes. The range of socio-economic status (SES) of the families was very wide as indexed by parental education and occupation. Parental education was coded on a six-point scale based on the level of education completed by each parent; zero = no schooling; one = primary school; two = middle-school; three = high-school or professional school; four = university degree; five = post-graduate studies. Occupation was coded on a scale of zero to two; zero = no occupation or not paid job; one = manual work; two = service sector or intellectual work. The level of education and occupation of the mother and the father were added into a single index of SES that ranged from 0 to 14. Families distributed according to the SES index in the following way: 1% were of high SES, 97.2% of middle SES, 1.8% of low SES. All parents provided informed consent for the tests and interviews. The data of all participants were collected in accordance with the Helsinki Declaration II and the Institutional Ethics Committee of the Psychology Department at Padova University.

### General procedure

Children completed a battery of numerical tasks, including tasks relying on approximate representations: magnitude comparison (Halberda et al., 2008) and number line task (Siegler and Booth, 2004); tasks relying on exact representations: counting (Geary et al., 1992), one-to-one correspondence (Van Luit et al., 1994); and a task assessing children's ability to solve math problems in everyday situations. Moreover an assessment of verbal short-term memory (digit span) was used as a covariate. All the tests were administered individually in a quiet classroom during school hours. A brief description of each of these tasks is presented in the following section.

Additionally, we obtained data about family activities from two questionnaires -one for the child and one for the parents. The child's questionnaire (see Supplementary Material) was designed to directly assess the children's knowledge of numerical facts acquired in the family environment such as birthdates, number of siblings, phone numbers. Each correct response scored one point. The total scoring of the children's questionnaire corresponds hereafter to “numerical information learned at home.”

In order to assess congruency between parents' and children's accounts of the information children knew, we also interviewed the parents about number-related information, asking whether they thought their children knew this information. Each affirmative response scored one point. We also asked parents about the family constitution and background. Moreover, parents indicated the frequency on a weekly basis of number-related and non-related activities of the child (e.g., playing board games, videogames, etc.) and those involving the family (e.g., shopping, reading, tv watching, etc.). A five-point incremental scale was used for scoring frequency: does not carry out this activity, does it for about 1, 2, 3, 4 h, or more per week.

The criteria for including specific daily activities in the questionnaire derives from studies indicating that such activities facilitate academic performance and cognitive control more generally (i.e., reading –LeFevre et al., 2009-; practicing sports -Trudeau and Shephard, 2008; playing videogames –Dye and Bavelier, 2004), or directly enhance numerical performance (e.g., music –Spelke, 2008; playing board games –Ramani and Siegler, 2008; Siegler and Ramani, 2008; frequency of shopping and talking about money with children –LeFevre et al., 2009). The activities chosen also corresponded to the most frequent activities typically occurring in Italian families (not necessarily benefiting scholastic performance, such TV watching –Christakis, 2009), and activities mentioned in similar research (Anderson, 1998; Blevins-Knabe et al., 2000; LeFevre et al., 2009). Cronbach's alphas for the questionnaire applied to children and parents were 61 and 68, respectively. These values are fully comparable with measures of internal consistency found in previous research on numbers in everyday activities (Semenza et al., 2014). Agreement between parents' answers and children's actual knowledge was on average 75.11%, *SD* = 15.68.

### Numerical tasks

The tests were administered individually in a silent room. No feedback was given, only general praise and encouragement. A brief description of each test follows.

- Counting tests assessed the child's mastery of the number word. The children were requested to count forwards (from 1 to 10; from 4 to 10) and backwards (from 6 to 1; from 10 to 1). Children were instructed to count as fast as possible. One point was given for each correct sequence.
- One-to-one correspondence task was adapted from the Early Numeracy Test (ENT-part A, Van Luit et al., 1994). The task was composed of 5 tests designed to assess the children's ability to understand one-one relations between objects and numbers. For example, the child has 15 blocks and the experimenter shows a drawing representing two dice with 5 and 6. Then the experimenter asks: Can you put as many blocks on the table as are shown on the dice here? Each correct response scored one point.
- Magnitude comparison: children were presented (maximum 2 s) with two panels –one on the left and one on the right– that contained randomly arranged sets of squares of varying sizes. In each trial participants were asked to point as fast as possible to the panel that contained more squares, and were prevented for counting. There were six test trials randomized across participants (6:9, 5:6, 6:8, 6:9, 2:5, 8:9). One additional trial (1:2) was used for practicing before starting the test. Each correct response scored one point. This task was based on previous studies assessing approximate skills in children (Halberda et al., 2008).
- Number line task*:* On each trial a 20 cm long line was presented in the center of a white A4 sheet. Children were asked to point to the numbers 3, 5, 6, 7, 10, and 14 in the intervals 0–10 and 0–20. The ends of the lines were thus labeled on the left by *0* and on the right by either *10* or *20*. The order of presentation of the two intervals and the order of items were randomized across participants. Each trial was performed in a separate line. Before asking the participant to estimate the position of a number on the line (i.e., marking the line with a pencil), the experimenter ensured that the child was well aware of the interval size by pointing to the endpoints of the line and stating: “This line goes from 0 to 10 [20]. If here is 0 and here is 10 [20], where would you position X?” The number to be positioned in the line was orally presented. Experimenters were allowed to repeat the number as many times as needed. The accuracy of children's estimates on the number line was calculated using the percent absolute error. This measure is frequently used to measure non-numerical estimation in children (Siegler and Booth, 2004; Booth and Siegler, 2006; Berteletti et al., 2010; Sella et al., 2015). Lower absolute error indicates more accurate estimates.
- Everyday numerical problems consisted of seven tests meant to assess the children's ability to use numbers in situations of the daily living. The problems involved the application of basic arithmetic operations (e.g., could you show me two types of fruits or vegetables that together will make 7 pieces?), magnitude comparison (e.g., are there fewer lemons or fewer strawberries?), or money usage [e.g., “each one of these vegetables costs 1 euro. Could you bring me the box that contains the number of vegetables that will make you spend all this money (5 euros)?]. Each correct response scored one point.

### Data analysis

We first performed Pearson's correlation tests to determine the association between the number-related tasks and the child and family informal activities. To make sure that the relationships between those tasks were not mediated by a general cognitive or demographic factor, we computed partial correlations between the number-related tasks and the child and family activities, in which the impact of children's age, short-term verbal memory, and the SES of the family were controlled for.

Additionally, we used a stepwise regression procedure to determine which of the environmental variables predicted variation on each of the math cognition tasks. We included the numerical tests as the dependent variables and the knowledge of number information learned at home, short-term verbal memory, age, and SES of parents as potential predictors. Before running the regression analysis we checked for collinearity among predictors. Because there was a high collinearity (*K* = 72) we also performed a Principal component analysis to obtain a new set of uncorrelated predictors to feed the regression. Because the results of the regressions using PCA yielded virtually the same results of the regression that used the original predictors, only the latter are reported.

## Results

### Descriptive statistics

The mean percentage of errors was 14% (range 0–75%) in the counting task, 22% (range 0–80%) in the one-to-one correspondence task, 13% (range 0–67%) in the magnitude comparison task, and 25% (range 0–75%) in everyday numerical problems. For the number line task mean percent absolute error was 21 (range 11–37%). It is worth noting that the accuracy of estimation in the number line task is similar to the one reported in comparable age groups (interval 1–10, studied by Berteletti et al., 2010, Experiment 2 = 20%; interval 1–20 = 15%).

### Correlational results

The results of the Pearson's correlation analysis showed that certain early numerical abilities were intercorrelated (see Table 1). The strongest correlation was observed between the children's counting abilities and their capacity to solve everyday numerical problems (*r* = 0.45; *p* < 0.001). Counting was also correlated with one-to-one correspondence tasks (*r* = 0.32; *p* < 0.001), and negatively correlated with the percent of absolute error measured in the number line task (*r* = −0.31, *p* < 0.005). The children's ability to solve everyday numerical problems was also correlated with the one-to-one correspondence task (*r* = 0.29; *p* < 0.005) and the magnitude comparison test. The latter correlation however disappeared after controlling for the children's age and verbal short-term memory, and the SES of the family.

**Table 1:** **Correlations coefficients obtained after correcting by age, memory span, SES of the family (Bold ***p*** < 0.001; bold and italics ***p*** < 0.005)**.

		**1**	**2**	**3**	**4**	**5**	**6**	**7**	**8**	**9**	**10**	**11**	**12**	**13**	**14**	**15**	**16**	**17**	**18**	**19**
**TASKS**																				
1	Counting	**–**																		
2	Magnitude comparison	0.22	**–**																	
3	Everyday numerical problems	**0.45**	0.21	**–**																
4	Number line	***–0.31***	–0.09	–0.23	**–**															
5	One–one correspondence	**0.32**	0.18	***0.29***	–0.01	**–**														
**CHILD'S KNOWLEDGE OF NUMBER RELATED INFORMATION**																				
6	Child's answers	***0.28***	0.12	**0.34**	0.06	***0.28***	**–**													
7	Parent's answers	0.16	0.06	0.02	0.09	0.19	**0.33**	**–**												
**CHILD DAILY ACTIVITIES**																				
8	Videogames	–0.11	–0.11	–0.14	0.01	–0.07	0.02	–0.05	**–**											
9	Reading	–0.09	–0.10	–0.25	0.08	–0.01	0.02	***0.29***	–0.02	**–**										
10	TV watching	–0.02	–0.15	–0.19	0.06	–0.14	–0.07	0.14	**0.32**	0.15	**–**									
11	Sports	–0.03	–0.06	–0.03	–0.05	–0.18	0.06	0.13	0.07	0.15	–0.05	**–**								
12	Music	0.02	–0.07	0.06	0.09	–0.03	0.11	0.08	–0.10	–0.04	–0.13	0.02	**–**							
13	Other activities	0.20	0.14	0.02	–0.19	0.18	–0.02	0.13	–0.13	0.27	0.08	0.24	–0.11	**–**						
**FAMILY DAILY ACTIVITIES**																				
14	Shopping	–0.23	–0.02	–0.18	0.16	0.05	0.03	0.21	0.11	0.13	–0.08	–0.02	–0.07	0.00	**–**					
15	TV watching	–0.08	–0.08	–0.20	0.10	–0.22	–0.13	0.03	0.27	–0.07	**0.26**	0.10	–0.21	0.01	0.17	**–**				
16	Reading	0.06	0.13	0.00	0.02	–0.01	–0.01	0.13	–0.13	0.23	–0.17	0.16	0.04	0.14	0.02	0.10	**–**			
17	Sports	0.03	–0.09	0.05	***–0.26***	–0.06	0.08	–0.01	0.08	0.02	–0.10	**0.44**	0.22	0.05	–0.02	–0.06	0.15	**–**		
18	Board games	***0.31***	0.04	0.18	0.09	0.19	***0.29***	**0.40**	0.12	0.11	0.02	0.17	0.00	0.01	0.03	–0.03	0.13	–0.01	**–**	
19	Videogames	–0.12	–0.12	–0.15	0.00	–0.09	0.01	–0.06	**0.99**	–0.03	**0.32**	0.06	–0.11	–0.15	0.11	0.28	–0.14	0.07	0.11	**–**

The children's responses to the questionnaire inquiring into the knowledge of number related information, and the responses of the parents to the same questions were positively correlated (*r* = 0.33; *p* < 0.001). Moreover, the numerical information learned at home significantly correlated with the children's performance on everyday numerical problems (*r* = 0.34; *p* < 0.001), counting (*r* = 0.28; *p* < 0.005), and the one-to-one correspondence task (*r* = 0.28; *p* < 0.005).

Considering specific activities within the family environment, we found a positive correlation between counting and the frequency with which children played board games at home (*r* = 0.31, *p* < 0.005). The frequency of playing board games also correlated with the numerical information learned at home as reported both by the parents *r* = 0.40, *p* < 0.001 and the children *r* = 0.29, *p* < 0.005. The frequency of playing sports in family negatively correlated with the percent of absolute error measured in the number line task (*r* = −0.26, *p* < 0.005). Other activities did not correlate significantly with the numerical tasks.

### Modeling numerical abilities

**Figure 1:**
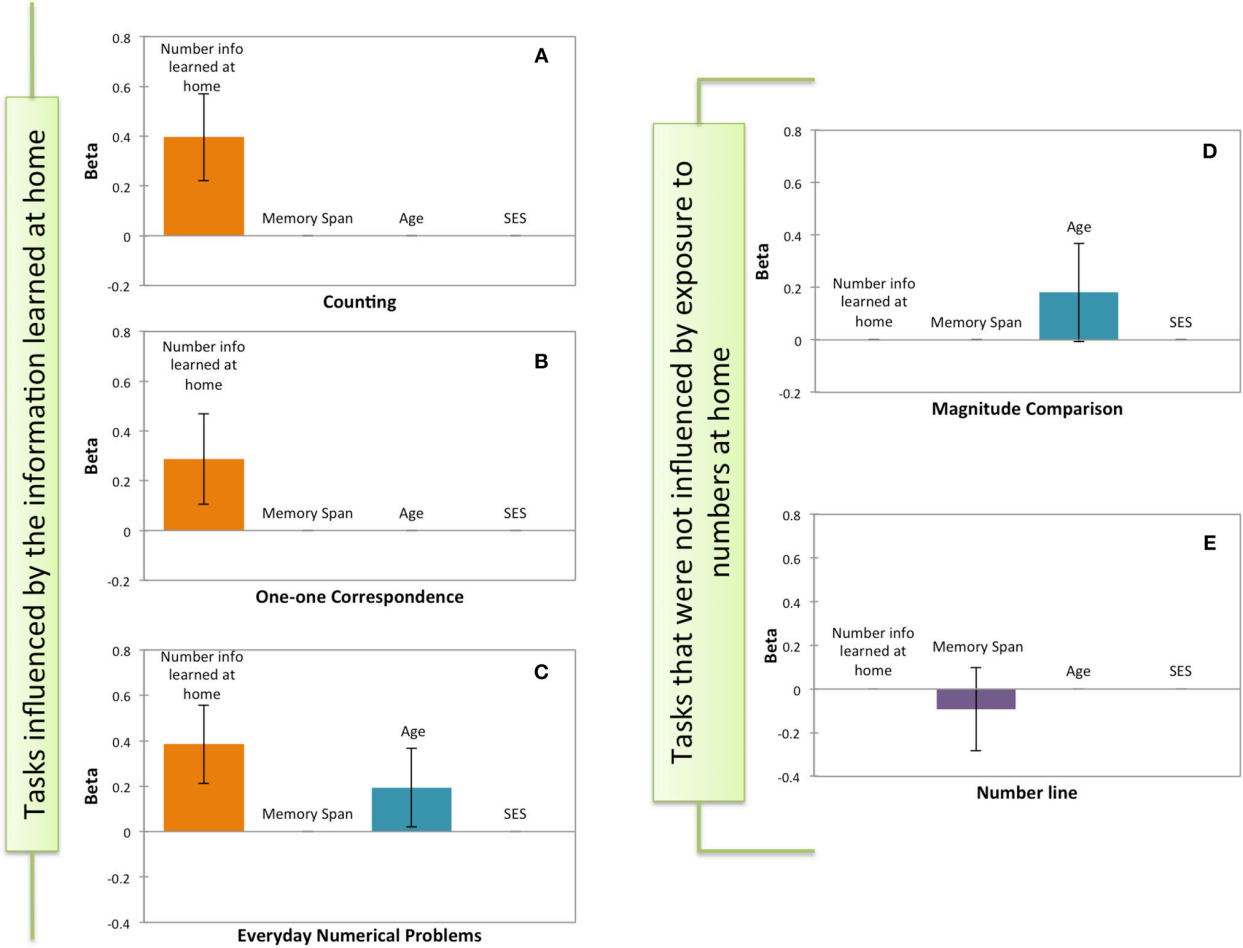
**Significant predictors of the stepwise regressions performed on each mathematical test**. **(A)** Counting. **(B)** One-to-one Correspondence. **(C)** Everyday numerical Problems. **(D)** Magnitude Comparison. **(E)** Number line test. The y-axis depicts the standardized scores (β) of the predictors in each the model. The x-axis shows the potential predictors: Number Information Learned at Home, Memory Span, Age, and SES. Error bars indicate 95% confidence intervals.

The stepwise regression with the counting ability as the dependent variable settled on a final model that included only numerical information learned at home as the actual predictor [β = 0.397; *p* < 0.0001; Model *R*^2^ = 0.157, *F*_(1, 109)_ = 20.154; *p* < 0.0001; all other Betas *p*'s > 0.05; Figure 1A]. To avoid confounding between the predicted variable and the predictors, we excluded the counting task from the questionnaire of numerical information learned at home. We obtained virtually the same results as above. Numerical information learned at home was the only significant predictor in the final model [β = 0.352; *p* < 0.001; Model *R*^2^ = 0.091, *F*_(1, 109)_ = 11.885; *p* < 0.001; all other Betas *p*'s > 0.05].

Similarly, numerical information learned at home predicted children's performance on one-to-one correspondence tasks [β = 0.287, *p* < 0.002; Model *R*^2^ = 0.083, *F*_(1, 109)_ = 9.728, *p* < 0.002; all other Betas *p*'s > 0.05; Figure 1B] and on the everyday numerical problems (β = 0.385; *p* < 0.0001), a smaller but still significant contribution to this model was obtained with the variable age [β = 0.193; *p* < 0.029; Model *R*^2^ = 0.188, *F*_(1, 109)_ = 12.364, *p* < 0.0001]. No other variables predicted children's performance on the everyday numerical problems (Betas *p*'s > 0.05; Figure 1C). Furthermore, in the stepwise regression with children's performance on the magnitude comparison task as the dependent variable, a trend emerged for age as predicting factor [β = 0.181; *p* = 0.058; Model *R*^2^ = 0.033, *F*_(1, 109)_ = 3.676; *p* < 0.058; all other Betas *p*'s > 0.05; Figure 1D]. Nearly the same results were found after excluding the pair that could be solved with subitizing strategies (i.e., 2:5): (β = 0.103; *p* = 0.057; all other Betas *p*'s > 0.05). Moreover, when considering only the items that could be at the critical ratio for 5–6 year-olds (i.e., 5:6, 8:9), the factor age became a significant predictor in the magnitude comparison task [β = 0.304; *p* = 0.006; Model *R*^2^ = 0.064, *F*_(1, 109)_ = 7.690; *p* = 0.006; all other Betas *p*'s > 0.05] [see Halberda and Feigenson (2008) for critical rations at these ages]. Finally, none of the other variables predicted children's performance on the number line task [Model *R*^2^ = 0.009, *F*_(1, 109)_ = 0.938; *p* = 0.335; Figure 1E].

## Discussion

In the current study, we explored whether activities within the family environment and numerical information learned at home relate to preschoolers' performance on different numerical tasks. The main question was whether numerical instruction embedded in real-life settings influences tasks that depend on approximate representations, on exact representations, or both.

The findings indicate that the early acquisition of numerical information within the family environment significantly predicts the children's ability to solve numerical problems in everyday situations, counting abilities, and the skills for identifying one-to-one correspondences between sets. Crucially, not every numerical skill was related to the acquisition of numerical information at home. Children's performance on number line or magnitude comparison tasks, that essentially tap the approximate system, were not predicted by the amount of numerical information learned in the family environment.

These findings are in agreement with previous studies showing a relation between numerical instruction at home and children's math performance (e.g., LeFevre et al., 2009; Anders et al., 2012). These data also extend previous research in several ways. First, the results show that the children's performance in numerical tests relates to the acquisition of numerical knowledge of everyday facts (e.g., ages, birthdates, phone numbers, etc.), not just to direct mathematical instruction by parents, such as teaching how to count (Blevins-Knabe and Musun-Miller, 1996; Pan et al., 2006), or add (Huntsinger et al., 2000; LeFevre et al., 2002). Second, our study shows that numerical information learned at home also predicts the children's ability to solve simple word problems containing arithmentic operations that they have not formally learned at this age. Third, our findings suggest that numerical information learned at home affects tasks involving exact representations of number. By contrast, this knowledge does not affect tasks that depend on approximate representations, such as magnitude comparison and number line estimation. For magnitude comparison, Piazza et al. (2013) found that formal education is correlated with better performance in the Munduruku, an Amazonian tribe without words for exact counting. Park and Brannon (2013) found that training improved both comparison performance and maths ability. However, both of these studies used adult subjects. Another study of adults by Cappelletti et al. (2013), found that training improved magnitude comparison but the improvement did not transfer to arithmetic performance. Thus, it would seem that while the magnitude comparison is trainable, it does not get trained by numerical activities in the home in young children. The effect on exact number tasks are therefore not mediated by the approximate number system in our study.

The numerical information learned at home had a large effect on the counting ability of the children, and this appears fundamental to arithmetic learning. For example, Geary and colleagues showed that individual differences in first graders' counting abilities correlated positively with differences in their arithmetic proficiency (Geary et al., 1992). A similar pattern of results was found by Aunola et al. (2004) in a longitudinal study. The authors identified the counting ability at preschool age as a reliable predictor of mathematical achievement in first grade. Moreover Passolunghi et al. (2007) also identified counting skills at the beginning of primary school as a direct precursor of mathematical learning 6 months later. Furthermore, in Reeve et al. (2012), the ability to exactly enumerate sets in kindergarten predicted age-appropriate arithmetical attainment at every year until age 11, when their longitudinal study ended.

The fact that parents' answers regarding children's knowledge of information did not correlate well with their children's performance on the test is not at all surprising. It supports previous views suggesting that children's accounts might be more informative than parental ones. It has been argued that, particularly in the mathematical domain, parents may miss a lot of activities, those when they are busy with other things, or when the child is at some distance from the parent (Tudge and Doucet, 2004). Reliance on parental accounts, rather than direct observations of, or conversations with, children also has the disadvantage that children's own experiences are devalued (Hogan et al., 1999). It has been shown that parents are generally more concerned with specific aspects of their children's cognitive development than others. Their teaching in some domains might be genuinely incidental whereas for other domains they make deliberate efforts to teach their own children. Those activities on which the parents focus more are therefore reported more reliably. LeFevre et al. (2009) showed, for example that the frequency with which parents reported storybook reading is considerably higher than the frequency of playing board or card games. This focus could be due to the parents' personal preferences, their priorities, or both. In any case they seem to affect the parents' ratings about activities of the children in other domains.

The present data also show a positive correlation between the frequency with which children performed specific activities within the family environment and their numerical abilities. In particular the results showed a correlation between the frequency with which children practiced sports and their performance in the number line task, which might be due to the common involvement of spatial abilities in both activities. Moreover, there was a correlation between the frequency with which children played board games at home, their knowledge of number related information and their counting abilities. These results add to the body of literature on early numerical cognition, providing evidence that playing board games correlates with the development of numerical skills in children (Ramani and Siegler, 2008; LeFevre et al., 2009). Board games require children to remember numbers and to exercise the counting procedure, therefore contributing to the enhancement of this numerical ability. These results are partially consistent with a previous study of Ramani and Siegler (2008) addressing the effects of playing board games over numerical development. This study showed that a 2 weeks board game program improved children's counting abilities and also the children's performance in numerical magnitude comparison and number line estimation. Consistent with their results, we found a correlation between the frequency of board game playing and the children's counting abilities. However, our results showed no correlation between the frequency of playing board games at home and either magnitude comparison or number line estimation. This discrepancy might be due to the differences in the intensity and type of the exposure. Whereas Ramani and Siegler exposed children to an intensive training with a single game, our study focused on playing board games in the family environment (not solely games designed to improve number line estimation). Additionally, while Ramani and Siegler found these environmental effects on children from low-SES, our study grouped children coming from a wide range of socio-economic backgrounds. Thus, another possibility is that the SES of the families modulates the effects of board games over numerical representations, in the same way the SES modulates interventions over early language skills (Hurtado et al., 2008) and cognitive development (Turkheimer et al., 2003).

The current results showing differential correlations between numerical knowledge acquired at home and each of the two numerical systems, questions the possibility that the exact and the approximate representations are interconnected, at least in this particular group. If the exact representations linked to the approximate system, one would expect parallel effects on both approximate representations and exact representations. Instead, we found that children who knew more number-related information only showed better exact representations; the same association was not apparent or transferred to the approximate system. Results from previous studies are in agreement with these findings. Using highly controlled training programs for children on either exact or approximate skills, Obersteiner and colleagues showed that participants improved only the skill trained with no crossover effects (Obersteiner et al., 2013). Additionally, Mussolin et al. (2012) found an association between tasks depending on approximate number system and the symbolic abilities in 3- to 4-year-old children, but such association was absent in the group of 5- to 6-year-olds that was comparable to the age of children in our sample. It is thus possible that the interactions between the two representational systems may change across development. However, it is also possible that the links between the two representational systems are present at different ages but become evident only longitudinally. This is an open question that requires further exploration.

The question arises at to the limits of the information learned at home and activities within the family environment as potential modulators of the children's numerical skills. Are younger children, who generally spend more time at home, more susceptible to the numerical activities within the family environment than older children? Is there a critical period after which these activities do not impact significantly the children's representations of numbers? While the current study focused on 5- to 6-year-old children's numerical abilities, future studies with children at different ages should be able to establish whether, for instance, earlier numerical experiences can provide cascading advantages to the young math learner. A comprehensive exploration of the developmental trajectories of different numerical abilities (e.g., counting, estimation, magnitude comparison) and their interaction with everyday exposure to numbers should contribute to further clarify these issues. Such information will be also crucial for identifying temporal windows in which interventions for children at risk might be more effective.

To summarize, the present study sheds light on the way numerical information acquired at home links to numerical representations in 5–6 years-old children. It is pointed out that the development of certain basic numerical concepts is associated with the amount of numerical facts acquired at home and the frequency with which children carry out specific activities within the family environment. In particular, the data suggests that early mathematical concepts associated with exact—but not approximate—representations can be enhanced when learning numerical information takes place in real–life activities at home.

### Limitations

At this point of our research it is not possible to identify the directionality of the correlations between numerical information learned at home and children's performance on certain numerical tests. One possibility is that children with higher intrinsic mathematical abilities demand more numerical information from the family environment. However, these results could also suggest that the frequent use of numbers in familiar interactions enhances children's numerical knowledge. Although it seems likely that a regular exposure to numerical information boosts basic numerical understanding in young children, more research is needed to determine the directionality of the influences that occur between intrinsic numerical abilities and family factors.

Another limitation of the work regards the extent to which the numerical information learned at home interacts with the information acquired in other institutions. Although current pre-school programs in Italy are not concerned with teaching birthdates, age, phone numbers of the family members, number of brothers, etc. we do not exclude the possibility that other number related everyday knowledge might be consolidated in this environment.

Finally, we obtained information about the specific board games the children played at home. All Italian parents who reported their children playing board games mentioned numerical games such as “tombola,” “uno,” “carte,” but also non-numerical games such as memory or puzzles. The time invested in each of these types of games was not assessed, however this information might be important to determine the nature of the games that significantly influence numerical understanding.

## Author contributions

SB, BB, CS conceived and designed the work. SB, FB, and GA acquired the data and performed the analysis. SB, BB, CS, and DL interpreted the final results. All the authors contributed writing and revising the manuscript.

### Conflict of interest statement

The authors declare that the research was conducted in the absence of any commercial or financial relationships that could be construed as a potential conflict of interest.
