# Supplementary material for: Numerical Activities and Information Learned at Home Link to the Exact Numeracy Skills in 5–6 Years-Old Children
Source: Front Psychol. 2016 Feb 11;7:94. doi: 10.3389/fpsyg.2016.00094 (PMC4750023; doi:10.3389/fpsyg.2016.00094)
Supplement: Supplementary file 1 [file DataSheet1.pdf]

Appendices

A) Interview for Parents

The interview for parents included the following questions:

General information

1. Name of the child

2. Age in years and months

3. Number of brothers/sisters and their respective age

4. Occupation of the father

5. Occupation of the mother

6. Last degree/level of education obtained by the father

7. Last degree/level of education obtained by the mother

Health

8. Did the child or the mother had problems during the pregnancy? If yes, which ones?

9. Was the child born at term? If not, please indicate the number of weeks of the pregnancy.

10. Does the child have any important health problem? Was he/she healthy during the firsts years of life?

11. Does the child have any hearing problem diagnosed?

12. Does the child have any visual problem diagnosed? If yes, please specify if he/she uses glasses to correct the problem

Daily activities

13. Which one of these activities do you and your child do together?

(please specify how many times and hours per week)

a-Shopping

b-Watching TV

c-Reading

d-Practice sports (How many? which ones? Do they require scoring? Is the child able to keep the scores by him/herself?)

e-Play board games (How many? which ones?)

f- Play videogames (How many? which ones?)

g- Other (please specify)

14. How many hours and how many times per week does your child spend in the following activities (do not consider the time he/she spends with adults in this activities):

a- Watching TV

b-Reading

c-Practice sports (which ones? How many?)

d- Play videogames (How many? which ones?)

e- Play outside with other children

f-Play an instrument (How many? which ones?)

e-Play alone with toys

g- Other (please specify)

Knowledge of number related information

15. Which one of these activities is your child able to do without help? (only questions marked with asterisk were including in the scoring. Additional questions were used as fillers)

a-(*) Count until 10

b-(*) Count until 30

c-(*) Tell his/her age

d-(*) Tell his/her birthday by heart

e-(*) Tell his/her address

f- Measure his/her own height

g- Measure his/her own weight

h-(*) Dial a phone number

i- (*) Tell the current time

j- (*) Tell the current date/year

k- (*) Tell the number of brothers/sisters and their age

l- Indicate whether he/she is late for school or any other activity

m-Get the money necessary to pay by him/herself for instance a bus ticket/train ticket

n- Choose the correct bus/train

o-Take the initiative to save money

p- Organize his/her own money to be able to buy stickers/ice-cream etc.

q-Cross the street

r-Ride a bike

B) Questionnaire for Children

Only questions marked with asterisk were including in the scoring. Additional questions were used as fillers.

Hi_________ (name of the child)____________!

1. (*) Could you tell me how old are you?

2. (*) When is your birthday?

3. (*) Do you remember in which year you were born?

4. (*) Do you have brothers and sisters? How many? How old are they?

5. (*) Could you tell me the address of your house?

6. Do you know what day of the week is today?

7. (*) What is the year?

8. (*) Do you know what date is today?

9. (*) Can you tell me the time? (if the child does not have a watch show him/her one so that he/she can answer).

10. (*) Could you give me the phone number of your mother or father?

11. What is your favorite game?

12. (*) Ok, now could you please show me how you count? (stop the child at 30).

13. How many children are there in the class?
